# Applications of Distributed-Order Fractional Operators: A Review

**DOI:** 10.3390/e23010110

**Published:** 2021-01-15

**Authors:** Wei Ding, Sansit Patnaik, Sai Sidhardh, Fabio Semperlotti

**Affiliations:** Ray W. Herrick Laboratories, School of Mechanical Engineering, Purdue University, West Lafayette, IN 47907, USA; ding242@purdue.edu (W.D.); spatnai@purdue.edu (S.P.); ssidhard@purdue.edu (S.S.)

**Keywords:** fractional calculus, distributed-order operators, viscoelasticity, transport processes, control theory

## Abstract

Distributed-order fractional calculus (DOFC) is a rapidly emerging branch of the broader area of fractional calculus that has important and far-reaching applications for the modeling of complex systems. DOFC generalizes the intrinsic multiscale nature of constant and variable-order fractional operators opening significant opportunities to model systems whose behavior stems from the complex interplay and superposition of nonlocal and memory effects occurring over a multitude of scales. In recent years, a significant amount of studies focusing on mathematical aspects and real-world applications of DOFC have been produced. However, a systematic review of the available literature and of the state-of-the-art of DOFC as it pertains, specifically, to real-world applications is still lacking. This review article is intended to provide the reader a road map to understand the early development of DOFC and the progressive evolution and application to the modeling of complex real-world problems. The review starts by offering a brief introduction to the mathematics of DOFC, including analytical and numerical methods, and it continues providing an extensive overview of the applications of DOFC to fields like viscoelasticity, transport processes, and control theory that have seen most of the research activity to date.



**Contents**
1      Introduction22      Mathematical Background4
2.1   Definitions and Properties...................................................................................................................................... 4
2.2   Distributed-Order Differential Equations................................................................................................................7
2.3   Solution of DODEs: Analytical Methods...............................................................................................................8
2.4   Solution of DODEs: Numerical Methods...............................................................................................................9
         2.4.1      Numerical Integration of the Integral Operator (Step 1)...........................................................................9
         2.4.2      Approximation of the Multi-term Fractional Derivatives (Step 2)............................................................103      Relevance of Distributed-Order Operators144      Applications to Viscoelasticity15
4.1   Constitutive Models............................................................................................................................................. 15
         4.1.1      DO Integral Models................................................................................................................................16
         4.1.2      Multi-Term Fractional Models................................................................................................................17
4.2   Material Characterization: Methods and Experiments.............................................................................................18
4.3   Distributed-Variable-Order Models........................................................................................................................18
4.4   Some Practical Applications.................................................................................................................................. 195      Applications to Transport Processes20
5.1   Anomalous Diffusion Processes.............................................................................................................................. 21
5.2   Reaction–Diffusion Processes................................................................................................................................ 24
5.3   Advection-Diffusion Processes............................................................................................................................... 25
5.4   Wave Propagation................................................................................................................................................. 256      Applications to Control Theory26
6.1   DO Controllers and Filters.................................................................................................................................... 27
6.2   Stability and Control of DO Systems.....................................................................................................................287      Conclusions29References30


## 1. Introduction

Fractional calculus (FC) was first introduced as a mathematical generalization of integer-order integration and differentiation. Started in 1695 from a discussion between Leibniz and de L’Hôpital about the possible interpretation of the operator $d^{n}/dx^{n}$ when n=1/2 [1], FC has been the object of studies for more than 300 years. In the early years, research mostly focused on mathematical aspects of the fractional-order operators; their physical interpretations and potential applications followed much later. Likely, the first application of FC can be traced back to Abel in 1826. Abel [2] applied FC to formulate an integral equation describing a tautochrone problem. Following Abel’s study, the integral representation of FC started gaining increasing attention in the mathematics community. Early works mostly focused on the development of analytical formulations to solve selected mathematical problems. The most immediate result of this rapidly growing interest in FC was the expansion of the possible definitions of a fractional operator including, but not limited to, the integral representation (Liouville, Riemann, and Hadamard) and the convergent series representation (Grünwald and Letnikov). While these early studies had pointed out the intriguing role that FC can play when modeling complex processes in physical systems, the bulk of the early research kept focusing on the development of the mathematical framework [3] and on the integration of these operators into ordinary and partial differential equations [4]. It was only in the second half of the twentieth century that the concept of FC started percolating to fields other than mathematics. An area of application that has seen a remarkably rapid growth is that involving the modeling of complex physical phenomena. Unlike integer-order operators, the intrinsic multiscale nature of fractional operators enabled a very unique and effective approach to model historically challenging physical processes involving, as an example, nonlocality or memory effects. Indeed, many of the early applications of FC to physical modeling included viscoelastic effects [5,6,7,8,9,10,11,12], nonlocal behavior [8,12,13,14,15,16,17,18,19,20,21,22,23,24], anomalous and hybrid transport [9,10,11,24,25,26,27,28,29,30], fractal media [12,31,32,33,34,35], and even control theory [36,37,38,39]. The interested reader is referred to the work in [40] for a detailed account of the birth and evolution of fractional calculus.

For more than a century, the study of fractional calculus focused on operators accepting a constant and single-valued order; we will refer to these operators as constant-order operators in order to differentiate them from the distributed (but constant) order operators that will be introduced below. Despite constant-order operators being considerably more general than their integer-order counterpart, the constant and single-valued nature of the order still limits its ability to accurately capture certain complex phenomena whose underlying physics could either evolve in time or emerge as the result of the interplay of multiple orders. In relatively recent years, this observation led to the formulation of two remarkable and unique forms of FC operators, namely, the distributed-order and the variable-order operators. The latter definition accounts for operators whose order can be a function of either dependent (e.g., state variables of the system) or independent (e.g., space or time) variables and can change value following the evolution of the system. While this review does not focus on this class of operators, the interested reader is referred to the works in [41,42] for a detailed overview of the mathematical aspects and applications of variable-order operators.

Before proceeding further, we clarify the different acronyms that will be used in this review in order to refer to the different types of fractional-order operators. The single constant-order operators are denoted as “CO” operators, the distributed-order operators (with constant order distribution) are denoted as “DO” operators, and the variable-order operators are denoted as “VO” operators. While VO operators can certainly be single or distributed in nature, with the acronym “VO” we specifically refer to single variable-order operators. Distributed-variable-order operators, which will be introduced later, are denoted as “DVO” operators.

The distributed-order definition of the operator allows considering a superposition of orders and accounting for, as an example, physical phenomena such as memory effects in composite materials [43] or multi-scale effects [44]. A typical example that illustrates the capabilities of this class of operators is the mechanical behavior of viscoelastic materials having spatially varying properties [45]. Distributed-order fractional calculus presents a natural generalization of constant-order fractional calculus (COFC) by integrating the fractional kernel of CO operators over an extended range of orders. Given that the fundamental kernel of a CO operator is retained in the DO operator, DO operators inherit the fundamental properties of COFC, such as the ability to model nonlocality and memory effects, and further extend them to multiple coexisting orders. This latter argument can be interpreted as a superposition of the behavior captured by individual CO operators using different orders within a given range.

**Figure 1:**
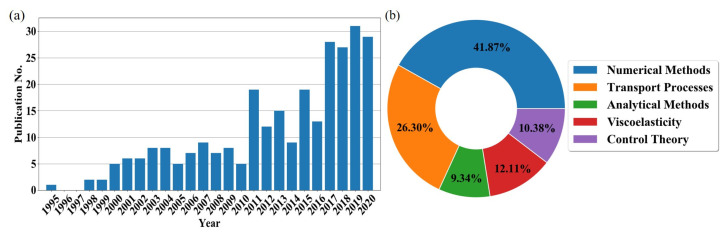
(**a**) Histogram chart showing the historical evolution of scientific publications per year starting from 1995. Note that the first study on distributed-order fractional calculus (DOFC) was published in 1966 by Caputo [46]. Approximately five studies were produced until 1995, which was taken as the starting year for the histogram. (**b**) Pie chart showing the distribution of publications per field. The data used in this figure were collected from Google Scholar.

The original concept of distributed-order fractional calculus (DOFC) can be traced back to the seminal studies by Caputo on dissipative elastodynamics [46,47,48]. In these studies, a generalization of the viscoelastic stress–strain constitutive laws, by employing a parallel sequence of fractional-order derivatives, was undertaken. Initially, the author dubbed this operator as the “*mean fractional-order derivative*”. A couple of decades later, Caputo [49] formalized the original proposition into the concept of DO derivative and also explored possible solutions to differential equations employing DO derivatives. Later, detailed investigations on the properties of DO operators, and on the properties and solution techniques of DO differential equations (DODE) were conducted in [45,50,51]. Following these pioneering studies on the mathematics of DO operators, in the 1990s and early 2000s, the interest in this topic went beyond the mathematical community and started percolating into several branches of engineering and physics. To date, we estimate that a total of approximately 300 papers have been published in the general area of DOFC. This estimate includes both journal and conference publications spanning a variety of fields including, but not limited to, theoretical and applied mathematics, analytical and numerical methods, viscoelasticity, transport processes, and control theory. A detailed time history and a quantitative assessment of the scientific studies produced in the general area of DOFC are provided in Figure 1.

Given the substantial critical mass reached by this field to date, and in view of the drastic acceleration of the research on DOFC observed in recent years, the time is ripe to assess the state of the field not only in terms of the mathematical formulation, but from the perspective of practical applications. In this review, we will provide a comprehensive discussion of the different fields of application and possible opportunities offered by DOFC to model complex physical problems. We expect that this review would serve as a starting point for the reader interested in approaching this fascinating field. Engineering, physics, chemistry, biology, and finance are only some of the communities that should find several points of interest and material for further consideration in this work.

The remainder of this paper is organized as follows. Section 2 focuses on providing an overview of the main mathematical concepts including basic definitions and properties of DOFC. The section also covers analytical and numerical methods for the calculation of DO operators and for the solution of DODEs. Section 3 briefly discusses the relevance of DO operators with respect to the modeling of complex physical processes. The remaining sections provide a review of the applications of DOFC to real-world problems including viscoelastic systems, transport processes, and control theory.

## 2. Mathematical Background

We begin this review by providing a brief summary of the basic definitions and properties of DO operators. Further, we will discuss the properties of differential equations with DO operators, and provide a brief overview of the corresponding analytical and numerical simulation techniques. We highlight here that, unless otherwise mentioned, the DO operator is defined on the basis of a general fractional-order derivative denoted by ${}_{c}^{□}D_{t}^{\alpha }$, evaluated with respect to a generalized independent variable *t*. We emphasize that the notation *t* used in this section must not be interpreted necessarily as time. Note that *c* denotes the lower terminal of the fractional derivative. The fractional derivative ${}_{c}^{□}D_{t}^{\alpha }$ can accept different definitions, although the most common for DO operators are those provided by Riemann–Liouville ${}_{c}^{RL}D_{t}^{\alpha }$ and by Caputo ${}_{c}^{C}D_{t}^{\alpha }$ [45]. Finally, also for the sake of brevity, we shall provide only the definitions corresponding to the left-handed fractional derivatives (the right-handed DO derivatives being an immediate extension).

### 2.1. Definitions and Properties

From a mathematical perspective, DO derivatives are defined as an integration of either the constant-order or the variable-order fractional derivatives with respect to the non-integer order of differentiation [48,49,50,51]. Two approaches to the definition of DO derivatives have been explored [45]. First, the so-called *direct approach* treats the order as a variable so that the DO derivative is defined as [45,49]

(1) $${}_{\alpha_{1},\alpha_{2}}D_{c,t}^{\alpha }\left(f\left(t\right),\kappa \left(\alpha \right),\alpha \right)=\int_{\alpha_{1}}^{\alpha_{2}}\kappa \left(\alpha \right){}_{c}^{□}D_{t}^{\alpha }f\left(t\right)\;d\alpha$$

where the integrand $\kappa \left(\alpha \right){}_{c}^{□}D_{t}^{\alpha }f\left(t\right)$ undergoes integration with respect to the independent variable α, that is, the fractional order within the interval $\alpha \in [\alpha_{1},\alpha_{2}]$. κ(α) is denominated as the order-weighting/strength function, or simply the strength function. The second approach, referred to as the indirect approach, treats the order as a function of a different independent variable *x* leading to the following definition [45],

(2) $${}_{x_{1},x_{2}}D_{c,t}^{\alpha (x)}\left(f\left(t\right),\kappa \left(\alpha \right),x\right)=\int_{x_{1}}^{x_{2}}\kappa \left(x\right){}_{c}^{□}D_{t}^{\alpha (x)}f\left(t\right)\;dx$$

where $x\in [x_{1},x_{2}]$ is the interval of integration. Similar to κ(α), κ(x) is also an order strength distribution [45]. The strength function (κ(α) or κ(x)) determines the contribution of each individual CO derivative to the overall DO derivative. As an example, a constant value of the strength function $\kappa \left(\alpha \right)=\kappa_{0}$ would mean the all the CO derivatives contribute equally to the final DO derivative [49]. The specific choice of this strength function depends on the underlying physics of the problem to be modeled and could be defined as either a continuous or a discrete function of the order α (direct approach) or the independent variable *x* (indirect approach). This latter comment is further clarified in the following section by using practical examples.

**Figure 2:**
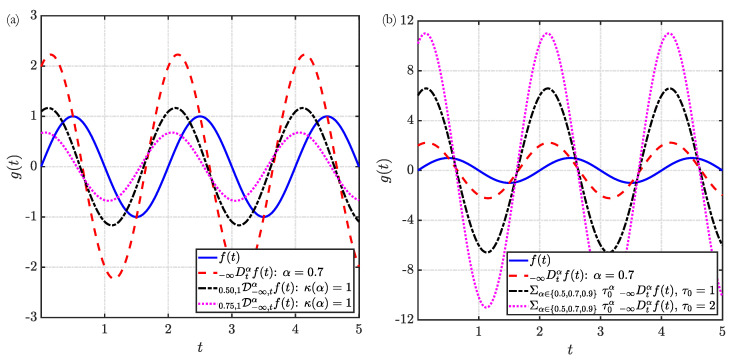
DO derivative of a harmonic function f(t)=sinπt derived following the definitions given in Equation (1). The plot shows the behavior of the derivative for (**a**) continuous and (**b**) discrete strength functions.

**Figure 3:**
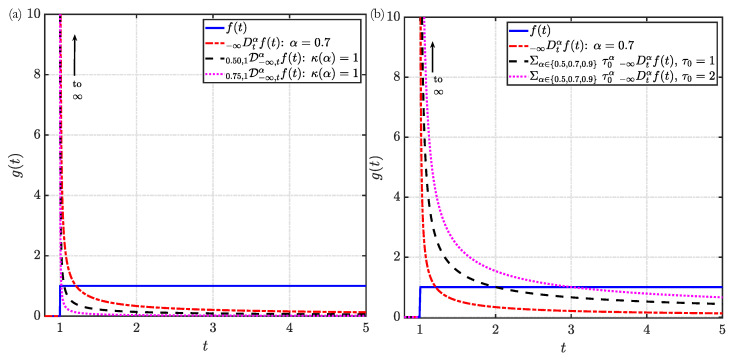
DO derivative of the Heaviside function f(t)=H(t−1) derived following the definitions given in Equation (1). The plot shows the behavior of the derivative for (**a**) continuous and (**b**) discrete strength functions.

To better illustrate the above concepts, we present a numerical demonstration of the DO derivatives evaluated for two representative functions of the variable *t*: (1) a sinusoidal function f(t)=sinπt in Figure 2 and (2) a step function f(t)=H(t−1) in Figure 3, where H is the Heaviside function. In Figure 2a and Figure 3a, the strength function is chosen to be κ(α)=1, such that it is constant and continuous. In the Figure 2b and Figure 3b, a discontinuous strength function $\kappa \left(\alpha \right)=\sum_{\alpha_{j}\in \left\{0.5,0.7,0.9\right\}}\;{\tau_{0}}^{\alpha }\;\delta \left(\alpha -\alpha_{j}\right)$, where $\tau_{0}$ is a positive constant. In generating the above results, we employed the Caputo definition of the fractional derivatives with terminals (−∞,t]. The CO Caputo fractional derivative of the two different functions to an order α∈(0,1) is [52]:

(3a) $${}_{-\infty }^{\;\;\;C}D_{t}^{\alpha }\left(\sin\pi t\right)=\pi^{\alpha }\;\sin\left(\frac{\pi \;(2t+\alpha )}{2}\right)$$

(3b) $${}_{-\infty }^{\;\;\;C}D_{t}^{\alpha }\left(H(t-1)\right)=H\left(t-1\right)\left[\frac{{\left(t-1\right)}^{-\alpha }}{\Gamma (1-\alpha )}\right]$$

The above CO derivatives are also provided in the Figure 2 and Figure 3 to facilitate comparison with the DO derivatives. Note that above expressions for the different CO derivatives identically reduce to their respective first-order (integer) derivatives for the choice of α=1.

As evident from the Figure 2 and Figure 3, the DO derivatives can be perceived as the weighted sum of individual CO derivatives over the specified range of fractional-order α. Particularly for κ(α)=1, as evident from Figure 2a and Figure 3a, the DO derivative is the linear sum of the CO derivatives with fractional-order α spanning the range $\left[\alpha_{1},\alpha_{2}\right]$. This concept is further illustrated by the examples in Figure 2b and Figure 3b. In these figures, the DO derivatives evaluated for $\tau_{0}=1$ are the sum of the individual CO derivatives. In contrast, for $\tau_{0}=2$ wherein the strength function is also a function of the order α, we observe a weighted contribution of the different CO derivatives to the DO derivative. The above discussion also explains the shift in the phase of the harmonic function in Figure 2a. More specifically, the phase shift in the DO derivative with respect to the original signal is caused due to the contribution of a phase difference of πα/2 (see Equation (3a)) by each CO derivative. The effect of the strength function on the amplitude, without changes in the phase, is illustrated in Figure 2b. Similarly, for the case of the Heaviside step function in Figure 3, different decaying characteristics can be obtained by varying the definitions of the strength function κ(α) and its support $\left[\alpha_{1},\alpha_{2}\right]$. Interesting applications to viscoelasticity based on this observation will be discussed in Section 4.

Lorenzo and Hartley [45] also extended the definitions of DO derivatives by allowing for the order distribution to be a function of different variables (such as, for example, space, time, or external loads). This extension introduced the concept of distributed-variable-order (DVO) operator. Following this extension, the direct and indirect approaches to the definition of DO operators can be reformulated as

(4a) $${}_{\alpha_{1},\alpha_{2}}D_{c,t}^{\alpha (t)}\left(f\left(t\right),\kappa \left(\alpha \right),\alpha \right)=\int_{\alpha_{1}}^{\alpha_{2}}\kappa \left(\alpha \right){}_{c}^{□}D_{t}^{\alpha (t)}f\left(t\right)\;d\alpha$$

(4b) $${}_{x_{1},x_{2}}D_{c,t}^{\alpha (x,t)}\left(f\left(t\right),\kappa \left(\alpha \right),x\right)=\int_{x_{1}}^{x_{2}}\kappa \left(x\right){}_{c}^{□}D_{t}^{\alpha (x,t)}f\left(t\right)\;dx$$

Although providing a very general form of the operator that can capture both multifractal (DO) and evolutionary (VO) behavior, the application of these operators has been rather limited. To date, most applications of DVO operators have been in the area of complex viscoelastic materials (see Section 4.3).

### 2.2. Distributed-Order Differential Equations

The present section is intended to briefly introduce the concept of differential equations based on DO operators. Clearly, the concept of DODEs is fairly extensive in itself and the reader is referred to the works in [53,54] for a detailed discussion on the different forms of DODEs and the corresponding solution techniques. Here, we simply introduce the general concept of DODE in order to facilitate the understanding of the discussion on applications presented in the remainder of the paper. Consider the following DODE [49],

(5) $${}_{0,m}D_{0,t}^{\alpha }\left(\kappa \left(\alpha \right),u\left(t\right),\alpha \right)=f\left(t\right)$$

for m∈N. Note that a discrete distribution function $\kappa \left(\alpha \right)=\sum_{j=1}^{n}b_{j}\delta \left(\alpha -\alpha_{j}\right)$ reduces the above equation to following multi-term fractional-order differential equation,

(6) $$\sum_{j=1}^{n}\;b_{j}\;{}_{0}^{□}D_{t}^{\alpha_{j}}u\left(t\right)=f\left(t\right)$$

At the same time, a continuous distribution κ(α)=C[0,m] can be perceived as a limiting case of the multi-term definition provided above when n→∞ [49]. While Equation (5) is an example of linear DODE, a nonlinear DODE can be given as [55]

(7) $$\int_{m_{1}}^{m_{2}}\kappa \left(\alpha \right)F\left({}_{0}^{□}D_{t}^{\alpha }u\left(t\right)\right)\;d\alpha =f\left(t,u(t)\right)$$

where $F\left({}_{0}^{□}D_{t}^{\alpha }u\left(t\right)\right)$ is a nonlinear function in the primary variable u(t) including its fractional derivatives.

For the linear DODE in Equation (5), some common assumptions are employed to ensure that the problem is well posed, that is, the solution is both bounded and convergent [55,56]:

**Hypothesis** **1.** *κ is absolutely integrable on the interval $\left[\alpha_{1},\alpha_{2}\right]$ and satisfies the following inequality,*

(8) $$\int_{\alpha_{1}}^{\alpha_{2}}\kappa \left(\alpha \right)s^{\alpha }d\alpha \neq 0,\;\;\;for\;\;Re\left(s\right)>0$$

**Hypothesis** **2.** *$f\in L^{1}\left[0,\infty \right)$, where $L^{1}$ is the Lebesgue space.*

**Hypothesis** **3.** *The function u(t) is such that ${}_{0}^{□}D_{t}^{\alpha }u\left(t\right)<M$$\forall t\in \left[0,\infty \right)\cap \forall \alpha \in \left[\alpha_{1},\alpha_{2}\right]$, where M is a constant. In other terms the fractional-order derivative is always bounded. For the limiting case where either of the order bounds tends to infinity (i.e., $\alpha_{1}\;or\;\alpha_{2}\to \infty$), the boundedness of the DO derivative requires the strength function κ(α) to be non-zero only over a finite range, that is, κ(α) must have a finite support [45].*

Pskhu [57,58] conducted early studies on the solvability of ordinary DODEs. Umarov and Gorenflo [59] extended these studies to analyze the solvability of multipoint problems. Diethelm and Ford [60,61] analyzed the existence and the uniqueness of solutions for linear DODEs, specifically for the case where Caputo-type initial conditions are available. Later, this proof was extended to the case where initial conditions are unknown [55]. It is noteworthy that these studies prove the existence and uniqueness for the fractional order α<1, while for α>1 the existence and uniqueness are still a conjecture. A similar exercise was performed on nonlinear DODEs with specific application to viscoelastic systems [62] and wave propagation [63]. The existence of solutions to hybrid DODEs was analyzed in [64], where the hybrid differential equations are quadratic perturbations to nonlinear DODEs [65,66]. Atanacković et al. also conducted similar studies on selected forms of DODEs encountered in the study of viscoelastic solids [67,68]. Note that all the aforementioned studies adopt the assumptions Hypothesis 1–3. Very recently, Fedorov studied linear DODEs that violate Hypothesis 2 resulting in an unbounded operator [69]. This study expanded the application of DODEs to initial and boundary value problems of ultra-slow diffusion.

### 2.3. Solution of DODEs: Analytical Methods

Concerning the analytical methods for the solution of DODEs, Caputo first proposed the use of Laplace transform to derive solutions [49]. Later, Bagley and Torvik [50,51] analyzed this approach in a systematic manner. The results obtained by the application of Laplace transform to DO derivatives are subject to minor modifications depending on the strength function and its support. Caputo derived the Laplace transform of DO derivatives with the order-distribution being an arbitrary interval [a,b]. Bagley and Torvik specialized this result for a restricted interval: α∈[0,1], given the numerous practical examples encompassed by this choice. Diethelm and Ford extended the domain to [0,m],m∈N [60]. The Laplace transform of a DO derivative with order distributed in [0,m], based on the Caputo definition, is given as [56]

(9) $$\begin{array}{ccc} L\underset{{}_{0,m}^{\;\;C}D_{0,t}^{\alpha }u\left(t\right)}{\underset{︸}{\left[\int_{0}^{m}\kappa \left(\alpha \right){}_{0}^{C}D_{t}^{\alpha }u\left(t\right)d\alpha \right]}}\; & = & \int_{0}^{m}\kappa \left(\alpha \right)\left(s^{\alpha }L\left[u\right]\left(s\right)-u\left(0\right)s^{\alpha -1}\right)d\alpha \\ \; & - & \sum_{j=1}^{m-1}\int_{j}^{m}\kappa \left(\alpha \right)u^{\left(j\right)}\left(0\right)s^{\alpha -j-1}d\alpha \end{array}$$

The Laplace transform of the DO derivative for other possible cases such as α∈[0,∞] and α∈[m−1,m] can be found in [45,70], respectively.

Using the Laplace transform of the DO derivative in Equation (9), Diethelm and Ford derived the analytical solution for the linear DODE: ${}_{0,m}^{\;\;C}D_{0,t}^{\alpha }u\left(t\right)=f\left(t\right)$ as [60]

(10) $$u\left(t\right)=u\left(0\right)+L^{-1}\left[\frac{1}{\int_{0}^{m}\kappa \left(\beta \right)s^{\beta }d\beta }F\left(s\right)\right]+\sum_{k=1}^{m-1}y^{k}\left(0\right)L^{-1}\left[\frac{\int_{0}^{m}\kappa \left(\beta \right)s^{\beta -k-1}d\beta }{\int_{0}^{m}\kappa \left(\beta \right)s^{\beta }d\beta }\right]$$

where $L^{-1}$ is the inverse Laplace transform. Note that the inverse Laplace transform in the above solution can be applied *iff* the assumptions Hypothesis 1–3, that ensure a bounded solution, are satisfied [60]. Lorenzo and Hartley derived analytical solutions for DODEs employing DO derivatives specifically for an order distributed over $R^{+}$ [45]. Other common approaches to derive solutions of DODEs include the Fourier method [71,72,73], the use of Mittag–Leffler functions [74,75,76], the spectral representation of the fractional operator [77], and series expansion methods [78,79]. The method of Laplace transforms combined with series approximations using Laguerre polynomials was also used to solve linear and nonlinear DODEs [80]. While the work in [80] focuses on obtaining the solution for one- and two-term fractional-order relaxation equations, the method developed in [80] is highly general and may be extended to DODEs with general strength functions.

Although, in the above discussion we have primarily considered DO derivatives based on the Caputo definition, the Laplace transform of DO derivatives based on the Riemann–Liouville definition can also be derived analogously [60]. In fact, as shown in [60], the only difference appears in the terms consisting the initial conditions, similar to the CO case [4]. This difference in behavior was also highlighted by Mainardi et al. [81], who employed Laplace transforms to compare the asymptotic behaviors of fundamental solutions to time-fractional DO diffusion equations. Interestingly, different asymptotic behaviors are observed for DO derivatives based on the Riemann–Liouville and Caputo definitions. The difference in the asymptotic behaviors is primarily due to the difference in the way the initial conditions appear in the Laplace transform of the CO Riemann–Liouville and Caputo derivatives [4,82].

### 2.4. Solution of DODEs: Numerical Methods

Although analytical solutions are possible for special types of DODEs [45,60], the rapidly growing application of DOFC to model complex physical systems often requires the use of numerical methods. Starting from basic observations, Diethelm [83] first proposed an approximate numerical method for the solution of multi-term DODEs. Following this initial study, several other numerical methods have been developed. Note that DODEs (see, for example, Equation (5)) can be either ordinary differential equations (ODE) or partial differential equations (PDE), depending on the specific application. The numerical simulation of either a distributed-order ODE or PDE requires the numerical approximation of the DO derivative. Once the approximation of the DO derivative is obtained, the procedure to numerically simulate the DODE follows exactly from classical procedures developed for integer-order equations. In other terms, the main difference between the evaluation of classical integer-order differential equations and DODEs lies in the numerical approximation of the DO derivative. In the interest of brevity, we focus this section only on this latter aspect. In general, the procedure to numerically approximate DO derivatives can be seen as a two-step process:

1. *Step 1*: Numerical integration of the integral operator. The DO derivative consists of a continuous distribution of the fractional order α. In Step 1, a numerical integration is used to discretize the DO derivative into a multi-term CO fractional derivative.
2. *Step 2*: Approximate solution of the multi-term fractional derivative. Following the conversion of the DO derivative into a multi-term fractional derivative at step 1, different numerical methods are used to evaluate each CO fractional derivative within the multi-term derivative.

The above two steps can be more practically visualized by considering the following example of DO derivative,

(11) $$\int_{a}^{b}\phi \left(\alpha \right)D^{\alpha }u\left(t\right)d\alpha \overset{\mathrm{Step}\;1}{\approx }\underset{\mathrm{Approximation of the integral}}{\underset{︸}{\sum_{i=0}^{k}W_{i}\phi \left(\alpha_{i}\right)D^{\alpha_{i}}u\left(t\right)}}\overset{\mathrm{Step}\;2}{\approx }\underset{\mathrm{Incorporate}\mathrm{approximation}\mathrm{of}\;D^{\alpha_{i}}u\left(t\right)}{\underset{︸}{\sum_{i=0}^{k}W_{i}\phi \left(\alpha_{i}\right)\Psi \left(\alpha_{i},t\right)}}$$

where $W_{i}$ is the weight obtained from numerical integration and $\Psi (\alpha_{i},t)$ is the numerical approximation of the CO derivative $D^{\alpha_{i}}u\left(t\right)$. In summary, at step 1, an approximation of the order integral is computed (often by quadrature rules), and at step 2, the remaining CO derivatives are approximated by employing different types of numerical methods for CO fractional derivatives. Based on this two-step approximation strategy, this section is divided into three parts: (1) a discussion of the most popular quadrature rules for the implementation of step 1, (2) a discussion of the various numerical methods for the implementation of step 2, and (3) a brief discussion on their computational aspects.

#### 2.4.1. Numerical Integration of the Integral Operator (Step 1)

As highlighted in the previous sections, a key difference between DO derivatives and CO derivatives is the existence of an additional integration over the order. To transform the integral form into the multi-term form (first of the two-step process), two common quadrature rules are often used by researchers: (1) Gauss–Legendre quadrature rule and (2) Newton–Cotes quadrature rule. Based on the Gauss–Legendre quadrature rules [84,85,86,87,88,89,90,91,92,93,94,95,96,97,98,99,100,101,102,103,104,105,106,107], the DO derivative can be approximated using the following multi-term form,

(12) $$\int_{a}^{b}\phi \left(\alpha \right)D^{\alpha }u\left(t\right)d\alpha =\int_{a}^{b}g\left(\alpha ,t\right)d\alpha =\sum_{i=0}^{k}W_{i}^{G}g^{G}\left(\alpha_{i}^{G},t\right)+R^{G}$$

where $W_{i}^{G}$ are the weights at the Gauss points $\alpha_{i}^{G}$ chosen for this integration over the DO. Although the Gauss–Legendre quadrature schemes are known to achieve highly accurate results (particularly when dealing with integrands of specific type such as, for example, polynomials), an analysis of the numerical convergence and of the truncation error (including steps 1 and 2) becomes difficult when the integrand consists of fractional derivatives (like $D^{\alpha }u\left(t\right)$, as shown in Equation (11)). To overcome these drawbacks of the Gauss–Legendre quadrature, the Newton–Cotes scheme was considered. The Newton–Cotes quadrature scheme can be divided into closed and open approaches, depending on whether the function values at the end points are included. Following the closed approach, different quadrature rules used for DO derivatives include the trapezoid rule [56,87,106,108,109,110,111,112,113,114,115,116,117], the Simpson’s rule [87,106,111,112,116,117,118,119,120,121], and the Boole’s rule [122]. All these schemes are also associated with different orders of convergence. Following the open Newton–Cotes approach, the mid-point rule is widely used [107,123,124,125,126,127,128,129,130,131,132,133,134,135,136,137,138,139,140,141,142,143]. The truncation error at the end of step 1, when employing the Newton–Cotes approach, simply follows the classical results. More specifically, the truncation errors are $O(h^{2})$ for trapezoid rule and mid-point rule, $O(h^{4})$ for Simpson’s rule, and $O(h^{6})$ for Boole’s rule. Given the flexibility in choosing different approximations and the ease of error analysis, Newton–Cotes method is typically preferred over Gauss–Quadrature approach in step 1 approximation.

#### 2.4.2. Approximation of the Multi-term Fractional Derivatives (Step 2)

As described in Equation (11), the second step involves the numerical approximation of the CO fractional derivatives within the multi-term fractional derivative. Strictly speaking, this approximation directly follows the techniques available for CO derivatives. The literature on numerical methods for the approximation of CO derivatives is extensive and has been the object of books [144] and papers [145,146,147]. Therefore, for the sake of brevity, we do not review again these methodologies.

The more interesting and challenging aspect, in the context of the DO formulation, is the combination of the step 2 approximation with the spatial and/or temporal discretization of the domain in order to develop computational models for space- and/or time-fractional DODEs. The different discretization techniques can be generally divided into (1) mesh-free approaches and (2) mesh-based approaches. The majority of mesh-free approaches are based on the spectral method, which uses basis functions to approximate the multi-term DO expression obtained in the first step. On the other hand, the mesh-based approaches involve most of the classical methods for differential equations including the finite difference method (FDM) and the finite element method (FEM). Depending on the specific implementation, that is, on the numerical technique adopted to approximate the CO fractional derivative in step 2 and the spatial and/or temporal discretization of the domain, the computational approaches differ in their accuracy and computational cost. This review focuses on this latter aspect. In this regard, we report here the accuracy of each method, wherever available. In order to unify the expressions for convergence analysis of different methods, we will use τ, *h*, and σ to represent the step-sizes in time, space, and order, respectively.

##### Mesh-Free Approaches

In this section, we briefly describe the different mesh-free approaches available in the literature to numerically simulate DODEs. The majority of these techniques adopt the common strategy of converting the DODE into a system of algebraic equations using orthogonal basis functions. This allows formulating operational matrices which approximate the CO derivatives within the step 2 approximation. Depending on the strategy adopted to develop these matrices (or, equivalently, these algebraic equations) the different mesh-free approaches can be broadly categorized as Galerkin methods, collocation methods, and tau methods. A brief discussion on these methods and some other miscellaneous techniques is provided in the following.

1. *Galerkin spectral methods* can be divided broadly into two categories depending on the specific nature of the basis functions: (1) Galerkin spectral methods based on Legendre polynomials (GLSM) and (2) Galerkin spectral methods based on Jacobi polynomials (GJSM). GLSMs were proposed very recently in [92,118,125,143,148] to solve time-fractional DODEs. These were accurate to $O(\tau^{2-\beta })$ (where, β∈(0,1)). A few researchers combined the GLSM scheme with an alternating direction implicit (ADI) scheme to improve the accuracy to $O(\tau^{2}+\sigma^{2})$[98,139]. Numerical studies based on the GJSM approach can be found in [85,91,149]. Some interesting conclusions were presented in [150], which combined a s-stage implicit Runge–Kutta method in time and the GJSM/GLSM in space to solve time-space-fractional DODEs. They established that a convergence of O(s+1) in time could be obtained when employing an algebraically stable Runge–Kutta method with order *p* (p≤s+1). A few researchers have compared the performance of the GLSM and GJSM techniques in [90,150,151]. The results of these studies indicate that the specific basis functions do not drastically alter the computational performance.
2. *Collocation methods* require that the approximate solution satisfies the DODE at specific locations known as the collocation points. Similar to the Galerkin spectral method, various collocation methods have been developed starting from (1) Legendre basis (LCM) [100,134] and (2) Jacobi basis (JCM) [105,152]. Zaky constructed a LCM to solve both linear and nonlinear boundary value problems [100], and later extended this method to simulate initial value DODEs [99,153]. Results indicated that the convergence error decays exponentially with an increasing number of Gauss–Legendre points. Very recently, the LCM was extended by Xu [96] to develop a higher-order Legendre–Gauss collocation method for nonlinear DODEs. JCMs were developed in [101,102,152] to solve DODEs concerning different physical applications (such as, for example, transport processes and control). A majority of the above studies achieved either first or second-order accuracy. Recently, Abdelkawy [105] proposed a fourth-order accurate scheme for time-fractional DODEs (admitting only smooth solutions) while also achieving an exponential convergence rate. Besides the popular LCM and JCM, collocation methods based on other basis functions including, for example, the Chebyshev polynomials [129,154], fractional Lagrange polynomials [92], and the wavelet method [119], were also developed. Some interesting numerical techniques were developed by combining selected aspects of the different basis functions such as, for example, the fractional-order Chelyshkov wavelets [104]. Similar to the Galerkin spectral methods, it appears that the different basis polynomials in the collocation methods, do not drastically alter computational accuracy.
3. *Tau methods* also employ different basis functions similar to the Galerkin spectral method and collocation method. Tau methods for DODEs were first developed in [155,156] using shifted Chebyshev polynomials. Building on these studies, shifted Jacobi polynomials were adopted as basis functions in [157], and shifted Legendre polynomials were adopted in [103,158]. A detailed analysis of the results from these studies suggests that the accuracy and computational cost of simulating a given DODE using the tau methods are similar to the collocation and Galerkin spectral methods.
4. *Other mesh-free methods* based on the formulation of fractional-order operational matrices have also been explored to solve DODEs. The operational matrix is based on different functions such as the block-pulse function (BPF) [89], Chebyshev polynomials [159,160], and shifted Legendre polynomials [154]. Following the same strategy, hybrid approximation methods based on the combination of different basis functions have also been developed. The specific combinations that have been explored in literature are BPFs and Bernoulli polynomials [95], BPFs and Taylor polynomials [93], and BPFs and shifted Legendre polynomials [161]. For completeness, we mention that other numerical methods including the Laguerre spectral method [108], Legendre wavelets method [84], fractional pseudo-spectral method [162], reproducing kernel method [163], radial basis function based mesh-free methods [86,114], and element-free Galerkin method [106] have also been proposed. Further, several semi-analytical approaches including the Homotopy perturbation method [164,165,166,167], harmonic approximations [168], and the Adomian decomposition method [169,170,171] have also been proposed and applied to derive the solution of DODEs and multi-term fractional differential equations (FDE).

##### Mesh-Based Approaches

Although many mesh-free approaches can be implemented relatively easily for DO problems involving simple geometries and boundary conditions, algorithms for numerical computations on complex domains (e.g., involving irregular geometry and high-dimensional systems) still present several complexities. This also reflects from the fact that many 2D and 3D problems have been solved using mesh-based approaches, while a majority of mesh-free approaches focus primarily on 1D problems. FEM is particularly useful in exploring numerical solutions over irregular domains. Among the mesh-based approaches for DODEs, two methods have generated the most interest: finite difference methods (FDM) and finite element methods (FEM). Before proceeding to review these mesh-based approaches, it is important to note a specific challenge faced by this class of methods. More specifically, due to weak singularity of the integral kernel within the fractional derivative, numerical solutions for initial boundary-value FDEs normally have non-smooth sharp approximations near the boundary [172,173,174]. As the DO derivative is approximated via a weighted sum of CO derivatives (see Equation (11)), this phenomenon also occurs when solving initial boundary-value DODEs [143]. To tackle this weak singularity, the commonly used mesh-based methods need to be improved. One possible approach, commonly adopted in literature, consists in the use of a graded mesh [87,143]. Remarkably, the use of the graded mesh also helps achieving a high-order convergence [87,143].

1. Finite difference methods are one of the most widely used mesh-based approaches for the solution of DODEs because they allow easy formulation and implementation. Compared with other approaches, the convergence and accuracy of FDM are easier to analyze [175,176,177]. A majority of the advanced FDMs are based on the Grünwald–Letnikov method (GLM) [122,142]. Recall that GLM uses a finite number of terms from a convergent series to approximate the fractional derivative and is a widely used approach [4]. Hu [126] used a shifted GLM to simulate a time-fractional DODE with accuracy up to $O(\tau^{1+\sigma /2}+h+\sigma^{2})$. Second-order accurate schemes for space-fractional DODEs were developed in [136] by using a Crank–Nicolson scheme in time and a shifted GLM. Similar second-order accurate algorithms can also be found in [133,178]. The second-order accurate backward difference formula, first proposed by Diethelm [145], also appears to be popular among several researchers [124,129,138]. To further improve the numerical accuracy, more elaborate methods were developed using the weighted and shifted GLM (WSGLM). Li [179] developed a numerical scheme with high spatial accuracy ($O(\tau^{2}+h^{4.5}+\sigma^{2})$) by combining WSGLM and the parametric quintic spline method. Another scheme capable of delivering high spatial accuracy ($O(\tau^{2}+h^{4}+\sigma^{4})$) was proposed by using the WSGLM for temporal approximation and high-order compact difference scheme for spatial approximation [117]. Yang [180] also proposed a similar composite method based on WSGLM in time and orthogonal spline collocation method in space. This scheme was shown to be unconditionally stable and accurate up to $O(\tau^{2}+h^{r+1}+\sigma^{2})$ (here *r* is the polynomial degree used in the spatial domain).
  FDM schemes have also been developed for high-dimensional problems, with particular attention being given to accuracy and convergence performance [141,181]. For applications requiring high accuracy, two techniques are often used: (1) compact FDM (CFDM) and (2) extrapolation method. Based on a fully discrete difference scheme [182], Ye [132] proposed a CFDM and demonstrated its convergence to be $O(\tau^{1+\sigma /2}+h^{4}+\sigma^{2})$. Pimenov [121] constructed a linearized difference scheme for nonlinear time delay DODE. Several researchers [110,120,183] also obtained a CFDM with order $O(\tau^{2}+h^{4}+\sigma^{4})$ based on higher order temporal approximation techniques. Gao [111,116] applied two extrapolation methods in time to achieve high temporal convergence: $O(\tau^{2})$ and $O(\tau^{2}|\ln\tau {|}^{2})$. For high-dimensional problems, ADI schemes become highly popular and help achieve highly accurate (second-order in time and fourth-order in space) numerical schemes [107,184].
2. Finite element methods: Starting from the study of multi-term FDEs, Jin [185] developed a Galerkin approach, Bu [186] used a multi-grid FEM, and Zhao [187] used a spatially nonconforming FEM to solve time fractional diffusion equations. Similarly, several researchers first developed FEMs to solve multi-term FDEs and later extended them to solve DODEs [87,123,188]. Few researchers [112,189] developed the $H^{1}$-Galerkin FEM for DO sub-diffusion equations which allowed the estimation of the diffusive field variable as well as its spatial derivative. By using locally discontinuous Galerkin FEM, Aboelenen [137] and Wei [190] developed highly accurate numerical schemes with spatial convergence $O(h^{k+1})$ (*k* is the degree of basis polynomials). Given the FEM’s unique ability of handling complex geometry, several recent studies have focused on its application to irregular domains. Examples include the development of FEMs, based on unstructured meshes, to solve DO equations corresponding to different physical applications [109,191,192,193].
3. Other mesh-based methods: In addition to FEM and FDM, a few other mesh-based methods were also explored. Examples include the combined B-spline interpolation and the Du Fort–Frankel method [130] for time-fractional DODEs. Heris [135] and Javidi [136] introduced a fractional backward differential formulas for space DODEs and obtained a second-order accurate numerical scheme. Diethelm et al. [60,188,194] introduced a convolution quadrature method for the numerical approximation of DO operators. Based on a backward difference formula, Podlubny [195,196] proposed a matrix form to represent discrete analogs of fractional operations and extended this method to the solution of DODEs [197]. Other mesh-based techniques developed in literature to solve DODEs and multi-terms FDEs include the predictor-corrector method [56,198,199,200,201] and the finite volume method [127,128,202].

##### Computational Aspects of DODEs

The previously discussed numerical schemes for the approximation of fractional derivatives typically generate dense matrices; a clear consequence of the intrinsic nonlocal character of the operator. For discretizations with *N* number of elements (temporal or spatial), these dense matrices generally require $O(N^{3})$ floating point operations and $O(N^{2})$ memory, for each iteration. In order to reduce this high computational cost, several alternate approaches were considered. Based on the idea of relabeling employed in ADI methods, Jia [203] developed a fast FDM which stores a coefficient matrix in O(N) memory and performs matrix-vector multiplication in O(NlogN) computations. Two numerical algorithms offering comparable time and space complexity were developed by Jian [142] and Zheng [202]. By expressing the matrix of coefficients as a sum of special diagonal-Toeplitz matrices, Jian derived a fast solution technique based on the preconditioned Krylov subspace method. Zheng proposed an efficient biconjugate gradient stabilized method to solve system of equations with a Toeplitz structured coefficient matrix. More recently, a reduced-order ADI method [184] was developed to reduce the computational cost involved in the numerical solution of DODEs.

Before proceeding further, it is worth noting that the computational time for the numerical simulation of DODEs can also be reduced via parallel computation and preconditioning of the operational matrices used to approximate the fractional derivatives. While parallel computation has not been directly applied to DODEs, parallel solvers have been developed for CO FDEs [204,205,206]. Besides the parallel algorithm itself, the effect of different hardware platforms (GPU v/s CPU) [207] and different memory architectures (shared memory v/s distributed memory) [206] on the computational times for simulation of CO FDEs, have also been studied. Further, preconditioners are often designed to accelerate matrix computations in nonlinear CO FDEs involving iterative problem solving procedures. Many studies have proposed different types of preconditioners such as, for example, preconditioned biconjugate gradient method [208] and generalized minimal residual method [209], for solving nonlinear CO FDEs. Both the above described techniques, that are parallel computing and preconditioning, present possible opportunities to reduce the computational time for solving DODEs and are hence worthy of detailed investigation in the future.

## 3. Relevance of Distributed-Order Operators

As evident from the definitions presented in Section 2, DO operators can be interpreted as a parallel distribution of derivatives of either integer or fractional orders. It follows that one of the most immediate application of these operators is to model physical systems whose response is characterized by a superposition of different processes operating in parallel and individually described by either fractional- or integer-order operators. As an example, consider electro-rheological fluids that can change their properties following the application of an electric field. This means that, in these media, the order of a small fluid element is dependent on the local field strength. Therefore, if the applied electric field is nonuniform, a corresponding order distribution will exist throughout the material [45]. A similar example consists of modeling the response of an electrical circuit with a distributed network of capacitors exhibiting the well-known fractional-order Curie’s law. According to this law, current through a capacitor varies with time *t* as $i\left(t\right)=V_{0}/Ct^{\alpha }$, where $V_{0}$ is a constant voltage and α∈(0,1) [210]. These simple examples suggest that there exists a class of physical problems that can be better described by DO operators.

Broadly speaking, the above-described class of physical problems is characterized by the presence of multifractal or equivalently multifractional systems [211]. The response of such systems is marked by the presence of multiple temporal and spatial scales, which can be accurately captured via time-fractional and space-fractional DO operators, respectively. The advantage of the DO operator in capturing the hierarchy of scales as well as anomalous scaling effects has been analyzed in detail in [44]. The occurrence of this hierarchy of scales could be better visualized by considering, for example, the modeling of turbulence via the Lévy walk approach. This approach associates a time scale with jump distances, and the multiplicity of scales is explicitly taken into account via an integral equation which contains a coupled memory kernel similar to the DO operator [212]. Other examples of such multifractional processes include the analysis of structures with simultaneous nonlocal and strain-gradient (multiscale) effects [213], diffusion of particles in microporous materials [214], analysis of financial markets where distributions of financial data usually possess fast falling power-law tails [215], and even state functions of complex quantum-mechanical systems [216,217].

From a different perspective, DO operators can also be used to retrofit models to experimental data derived from systems with an unknown fractional behavior. The fractionalization of differential equations commonly used in mathematical physics leads to the analysis of the order-parameter, say α, to be determined via experimental results. Ase experiments can lead to several values of the fractional order, as a result of different experimental conditions, it is convenient to introduce a DO fractional derivative. This is equivalent to integrating the product of a fractional derivative ($D_{□}^{\alpha }\left(\cdot \right)$) of the primary response variable (say *u*) and a weight function (or distribution) with respect to the order of the derivative, that is, to evaluate $\int_{\mathrm{supp}\;\phi }\phi \left(\alpha \right)D_{□}^{\alpha }ud\alpha$. In this way, one may use several experimental results and determine a continuous function ϕ rather than focusing on a single variable that is the fractional-order α. This can be interpreted as a homogenization of the different possible fractional processes and the resulting epistemic uncertainties. In other terms, such an approach would enable a valid and accurate analysis of experimental data and allow the development of fractional-order models, without having to identify the specific underlying fractional behavior.

The above remarkable properties of DO operators have led to the development of fractional models capable of describing numerous complex physical processes. Most of the work to date has concentrated on the general areas of viscoelasticity, transport processes, and control theory. We make a few concluding remarks, before proceeding to review the most significant applications of DOFC reported to date in the different areas. Note that the application of DOFC to viscoelasticity and control theory primarily involves the use of time-fractional DO derivatives, while the application to transport processes involve both space- and time-fractional DO derivatives. This separation follows from the underlying physics being captured. In this regard, recall that, while time-fractional DO derivatives are typically used to account for memory effects and dissipation across multiple temporal scales, space-fractional derivatives are used to model nonlocal effects and spatial heterogeneity over multiple spatial scales. In the applications presented below, we do not specify if the DO model is based on a Riemann–Liouville or Caputo (or any other) definition, as it marginally affects the overall discussion. Finally, we use the following notation in all the subsequent sections: *t* and *x* refer to the independent variables in time and space, respectively.

## 4. Applications to Viscoelasticity

Fractional-order derivatives are well suited to capture the dissipation in viscoelastic solids. The differ-integral definition of the fractional derivatives allows the effects of deformation history to be realized within the stress–strain constitutive models, thus combining the elastic response across different time scales. In this regard, Gemant [218,219], Caputo [46], Bagley and Torvik [5,6], and Chatterjee [7] provided seminal contributions towards the use of fractional-order models to simulate the effect of dissipation in viscoelastic solids. While an approach based on CO time-fractional derivatives is intuitive and has drawn much interest, it is not well suited for applications involving materials characterized by multiple relaxation times. In order to address this gap in modeling viscoelastic systems via the CO derivatives, DO models were proposed [48,49,220]. As mentioned in Section 3, the DO operators allow the multiple relaxation scales to be visualized as separate viscoelastic connections operating simultaneously. Thus, a superposition of multiple CO derivatives (or equivalently, multiple relaxation scales) is achieved via the definition of the DO derivative for viscoelastic solids.

### 4.1. Constitutive Models

**Figure 4:**
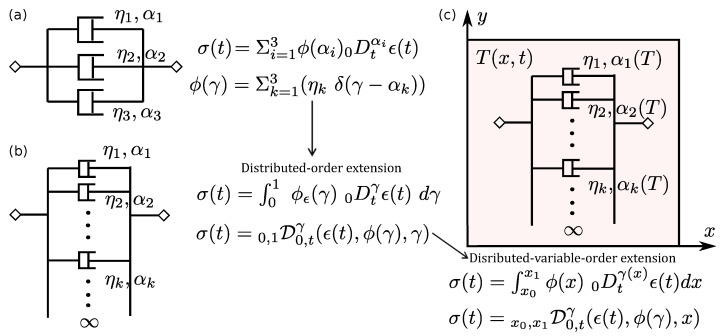
Examples illustrating the different DO models of viscoelasticity along with their respective constitutive relations. It appears that DO operators can model multiple viscoelastic elements within the same general formulation. Dashpots characterized by material constants η and order α indicate the individual viscoelastic elements. Schematic illustration of (**a**) the multi-term DO viscoelastic model, (**b**) the generalized DO model depicted as an infinite ensemble of elements with $\alpha_{i}\in \left(0,1\right]$ such that $\mathrm{Span}\;\left\{\alpha_{i}\right\}\;\mathrm{is}\;\left(0,1\right]$, and (**c**) the generalized temperature field-dependent VO definition for the DO viscoelastic model.

As mentioned in Section 2.1, the DO derivatives were originally conceptualized to model the dissipative elastic response with several temporal relaxation scales [48]. Following this seminal work, several other models of viscoelasticity either based on DO derivatives now exist in literature. These models can be viewed as simplified versions of the following generalized DO stress–strain constitutive law, proposed by Atanacković, for viscoelastic solids [221,222]:

(13) $$\int_{0}^{1}\phi_{\sigma }\left(\gamma \right){}_{0}D_{t}^{\gamma }\sigma \left(t\right)d\gamma =E\int_{0}^{1}\phi_{\epsilon }\left(\gamma \right){}_{0}D_{t}^{\gamma }\epsilon \left(t\right)d\gamma$$

where $\phi_{\sigma }$ and $\phi_{\epsilon }$ represent the strength functions corresponding to stress and strain (these are constitutive functions that characterize the viscoelastic response), *E* is the Young’s modulus, and ${}_{0}D_{t}^{\gamma }\left(\cdot \right)$ is the CO time-fractional derivative. The formulation in Equation (13) is referred to as the most general model because all other models, already existing in literature, can be derived from this model via suitable assumptions on the additional (fractional-order) constitutive parameters. For instance, the choice $\phi_{\sigma }=\delta \left(\gamma \right)$ and $\phi_{\beta }=\delta \left(\gamma -1\right)$ for the for strength functions results in the standard dashpot. Additional abstractions of the DO constitutive model in Equation (13), describing different viscoelastic elements, are illustrated in the Figure 4. Further, as discussed in Equation (6), a discrete choice for the order-distribution weights in Equation (13) would result in a multi-term fractional-order expression for the DO definition given above. Employing discrete strength functions in the above equation, the stress and its temporal derivatives (of real order, not necessarily integer) can be recast in terms of strain and its (real-order) temporal derivatives as follows [223],

(14) $$\sum_{n=0}^{N}a_{n}\left[{}_{0}D_{t}^{\alpha_{n}}\sigma \right]=\sum_{m=0}^{M}b_{m}\left[{}_{0}D_{t}^{\beta_{m}}\epsilon \right],\;\;\;\;t>0$$

where the fractional-orders are assumed to satisfy: $0\leq \alpha_{0}<\alpha_{1}\ldots <\alpha_{N}<1$, $0\leq \beta_{0}<\beta_{1}\ldots <\beta_{M}<1$. The constants $a_{□}$ and $b_{□}$ can be interpreted to be relaxation times for the viscoelastic solid. As demonstrated in [223], the above-presented multi-term model is effective in modeling both stress relaxation and creep response in viscoelastic structures. The integral constitutive relation given in Equation (13) can be interpreted as the continuum limit of the discrete multi-term constitutive relation given in Equation (14). This is also illustrated in Figure 4b, which depicts the DO integral model as the continuum limit of the discrete model in Figure 4a.

#### 4.1.1. DO Integral Models

All existing models catering to different lossy materials can be recast into the DO form in Equation (13) (or equivalently, Equation (14)) by considering different choices for order-distribution functions. In other words, each of the several distinct classifications of the viscoelastic solids proposed by Caputo and Mainardi [224] based on the creep and relaxation moduli relations, can be described by the single DO constitutive law via suitable choices of the fractional-order constitutive parameters. This highlights the relevance of DO operators and their scope in modeling viscoelastic constitutive relations when compared with other more classical integer—and fractional—(CO or VO) models available in the literature. To better illustrate this, consider the following two cases: case I: $\phi_{\sigma }=\delta \left(\gamma \right)$, $\phi_{\epsilon }=\tau_{0}^{\alpha }$, and case II: $\phi_{\sigma }=\tau_{\sigma }^{\alpha }$, $\phi_{\epsilon }=\tau_{\epsilon }^{\alpha }$, $\tau_{\sigma }<\tau_{\epsilon }$, $\tau_{□}$ being a material constant. These two choices for the integral forms of the DO constitutive relation are commonly used in modeling viscoelastic solids [43,225,226,227]. Depending on the choice of the strength functions, Equation (13) can successfully characterize both fluid-like and solid-like viscoelastic materials. Remarkably, salient mechanical characteristics of the viscoelastic materials modeled by these choices, such as the creep and stress relaxation functions, exhibit the experimentally observed power-law attenuation [228].

#### 4.1.2. Multi-Term Fractional Models

Compared to integral models, the discrete multi-term approach has been more widely used for the modeling of viscoelastic constitutive relations. This is a direct consequence of the simplicity with which discrete models could be modified in order to account for different lossy behaviors observed in real materials. The discrete form also facilitates a direct comparison between the viscoelastic behavior captured by DO models with respect to the more traditional and established integer-order models. This enables a better understanding of the physical relevance of DO models and it also allows a more natural approach to material characterization. The following instances of the different viscoelastic models that can be recovered from the multi-term DO law in Equation (14) further illustrate the strength of the DO approach:

1. *Kelvin-Voigt models:* The DO analogue of the Kelvin–Voigt model is obtained for the choice of $\phi_{\sigma }=\delta \left(\gamma \right)$, and $\phi_{\epsilon }=\tau^{\gamma }$ [229].
2. *Maxwell models:* The fractional-order Maxwell model of viscoelasticity can be obtained for $\phi_{\sigma }=\delta \left(\gamma \right)+\tau^{\alpha }\delta \left(\gamma -\alpha \right)$ and $\phi_{\epsilon }=E_{\infty }\tau^{\beta }\delta \left(\gamma -\beta \right)$ in Equation (13) [230]. Note that, assuming α=β in the fractional Maxwell model, allows recovering the fractional Zener model [231].
3. *Zener models:* If the material constants in Equation (14) are chosen as $a_{0}=b_{0}=1$, $a_{1}=a$, $b_{1}=b$, and orders $\alpha_{0}=\beta_{0}=0$, $\alpha_{1}=\beta_{1}=1$ the classical Zener model is obtained. Similarly, $\alpha_{1}=\beta_{1}=\alpha$ gives the generalized Zener model [232]. Wave propagation in fractional Zener-type viscoelastic media, obtained by choosing $\phi_{\sigma }=\phi_{\epsilon }=\delta \left(\gamma \right)+\tau^{\alpha }\delta \left(\gamma -\alpha \right)$ in Equation (13), was studied in [233,234]. Similarly, the choice of $\phi_{\sigma }=\delta \left(\gamma \right)+\left(a/b\right)\delta \left(\gamma -\left(\alpha -\beta \right)\right)$ and $\phi_{\epsilon }=a\delta \left(\gamma -\alpha \right)+c\delta \left(\gamma -\eta \right)+\left(ac/b\right)\delta \left(\gamma -\alpha -\eta +\beta \right)$ in Equation (13), also results in a fractional version of the classical Zener model with springs and dashpots [223].
4. *Other models:* Viscoelastic models described for the strength functions $\phi_{\sigma }=\delta \left(\gamma \right)+\tau^{\alpha }\delta \left(\gamma -\alpha \right)$ and $\phi_{\epsilon }=E_{0}\left(\delta \left(\gamma \right)+\tau^{\alpha }\delta \left(\gamma -\alpha \right)+\tau^{\beta }\delta \left(\gamma -\beta \right)\right)$ in Equation (13), were analyzed in [235]. Variations of this latter model (also referred to as the four-parameter model [236]) including the use of five-parameters [237] were studied to simulate selected types of lossy behavior in real materials. Further extensions that explored the use of additional terms were also presented [79].

In the above discussion, {a,b,c} denote different material constants corresponding to different relaxation times and {α,β,η} are the fractional-orders associated with different lossy behaviors of the DO model (see Equation (14)). In conclusion, we note that the multi-term fractional model is highly general and offers much flexibility in modeling different types of lossy behavior in viscoelastic solids. This is unlike CO or VO approaches that require separate models to capture these different behaviors.

### 4.2. Material Characterization: Methods and Experiments

It is clear from the discussion in Section 4.1 that several possibilities for the viscoelastic constitutive theories exist, considering suitable choices for the DO model parameters. Before proceeding to review the application of these DO theories to the characterization of viscoelastic materials, we make an important remark. Note that the application of these DO theories to real-world viscoelastic problems requires that these models are physically as well as mathematically consistent. To ensure consistency of the DO viscoelastic theories, there exist restrictions on the choice of the fractional model parameters which are derived in accordance with the principles of (1) time invariance, (2) causality, and (3) thermodynamics (dissipation inequality given by the Clausius–Duhem inequality) [49]. The conditions over the strength distribution functions $\phi_{\sigma }$ and $\phi_{\epsilon }$, corresponding to the integral definition of the DO law given in Equation (13), are available in [222]. For instance, the thermodynamic law restricts the choice of DO constitutive parameters for the fluid-like viscoelastic materials, discussed in Section 4.1.1, as follows, $\tau_{0}>0$. An analogous study conducted on the discrete form of the DO constitutive law (see Equation (14)) identified the restrictions on relevant constitutive parameters [223]. The investigations conducted in the aforementioned studies were further extended in [53] which analyzed the physical as well as mathematical consistency of the generalized DO model of viscoelasticity. In this regard, note that mathematical consistency ensures the existence and uniqueness of a linear viscoelastic response corresponding to the generalized DO formulation. The framework developed in [53] provides the foundation for a rigorous and consistent application of DOFC to modeling the response of viscoelastic solids.

The discussion in Section 4.1 highlighted the ability of DO operators to capture multiple scales of relaxation time and thereby different lossy behaviors observed in real materials [220]. For this purpose, the constitutive parameters of the DO constitutive model in Equation (13) that require to be identified are the fractional-order parameters and their numerical range. Initial investigations [82,220] laid a theoretical foundation for this fractional-order system identification problem. Further experiments on the characterization of viscoelastic properties corresponding to the different class of DO models for commercial polymers are reported in [238]. Such studies were carried out by matching the experimental profiles of the loss and storage moduli for viscoelastic materials [53]. Recall from Section 4.1.2 the relevance of DO operators in modeling multiple forms of viscoelastic behavior. This feature of the DO constitutive models for viscoelastic elements presents an interesting opportunity. To better illustrate this aspect, consider the multi-term DO models depicted in Figure 4a as the sum of several independent viscoelastic connectors with their associated relaxation timescales. This type of arrangement allows incorporating multiple timescales within a single DO model in order to design an optimized fractional damper. The incorporation of multiple timescales (using the DO derivative) can also be visualized from the DO derivative of the Heaviside step function in Figure 3. The relaxation time of the viscoelastic damper can be tuned by an appropriate choice of the constituent CO derivatives and their associated weights within the definition of DO derivative. This approach presents an opportunity to identify the damper that can deliver a desired behavior in terms of overshoot, peak time, and integrated tracking error [239]. This feature is unlike the classical integer-order or CO constitutive theories that allow only a single type of lossy behavior to be captured with a given model.

### 4.3. Distributed-Variable-Order Models

The above discussion presented an overview of the applications that DO models, based on CO derivatives, enable in the general area of viscoelastic solids. A few studies have also explored the extension of these models to employ DO operators based on VO derivatives; here below referred to as distributed-variable-order (DVO) operators. Lorenzo and Hartley presented one of the first works exploring the combination of both VO and DO operators to the formulation of the stress–strain constitutive law of viscoelastic solids [45]. They discussed how a DVO operator defined using a spatially-dependent VO law could be used to model the response of a thermorheologically complex material subject to a spatially and temporally varying temperature field. By choosing a spatially-dependent VO law, the resulting DVO model is capable of describing the spatial variation of the viscoelastic properties. The spatial variation of viscoelastic properties can be the result of a combination of internal as well as external conditions such as, for example, varying microstructure, presence of thermal loads, and a distribution of thermal gradients. We merely note that, very recently, this concept of defining a spatially-dependent VO law was used to model nonlocal solids with spatially varying microstructure in [240]. Further, an example of the temperature-dependent DVO viscoelastic model is illustrated in Figure 4c. In this case, the DVO model is required to introduce the effect of a spatially varying temperature field T(x,t) on the multiple timescales present within the DO model for viscoelasticity. This allows an accurate representation of the transient viscoelastic response [220]. It is important to mention that, unlike the DO models employing CO derivatives, the thermodynamic basis for the DVO models still remains to be ascertained.

### 4.4. Some Practical Applications

The DO constitutive models have been successfully applied in the analysis of viscoelastic solids. Recall that the different DO constitutive models can be classified primarily into two classes: (1) integral-models and (2) multi-term models, corresponding to the choice of DO derivative. Further, within each of these classes, further subdivisions exist depending on the specific functions chosen for (a) weights of the order-distribution functions and (b) bounds of the fractional-order α. Here, we shall present some prominent examples studied in literature that cater to a specific class of viscoelastic solids. These studies include finite solids with appropriate boundary conditions, and also the infinite solids.

Some examples of the constitutive parameters within DO integral models in Equation (13) were discussed previously in Section 4.1.1. Employing specific choices of the constitutive parameters, successful modeling of the creep response [225] and stress-relaxation [226] in finite solids is possible. Further, these integral models find relevance in modeling the vibration of fractional DO oscillators [227]. Patnaik and Semperlotti [168] demonstrated a successful application of DO viscoelastic models in the analysis of nonlinear oscillators with distributed nonlinear properties. In this study, the effect of the order-distribution on the phase and frequency response was captured analytically using asymptotic techniques and some important characteristics, such as simultaneous phase and amplitude modulation (that is not seen in integer-order models) were presented. Recently, the scope of DO constitutive models is also being explored to describe viscoelasticity within complex materials like composites [43].

These studies can also be extended to modeling and analyzing the damping of the structural response. DO models can be utilized to derive moment–curvature relations of viscoelastic rods [241,242,243]. The DO constitutive relation between moment ($\bar{M}$) and curvature ($\bar{\kappa }$) for the viscoelastic rod is given by

(15) $$\int_{0}^{1}\phi_{\bar{M}}\left(\gamma \right){}_{0}D_{t}^{\gamma }\bar{M}d\gamma =\int_{0}^{1}\phi_{\bar{\kappa }}\left(\gamma \right){}_{0}D_{t}^{\gamma }\bar{\kappa }d\gamma$$

In this equation, the choice of $\phi_{\bar{M}}=\delta \left(\gamma \right)$ and $\phi_{\bar{\kappa }}=EI\delta \left(\gamma \right)$ (EI is the bending modulus) reduces the above expression to the classical Euler–Bernoulli beam theory. The solution to the above DODE would reflect the influence of viscoelastic damping over the bending response of beams. Similar exercises can be conducted over more complex shapes with the help of advanced numerical techniques discussed in Section 2.4.

Employing the multi-term definition of the DO constitutive relations, the DO moment–curvature relations can be revisited for different classes of viscoelastic solids. For instance, DO bending relations analogous to the generalized Zener model were derived to study the dynamics of a viscoelastic rod in [243,244]. Similarly, the lateral vibration of a viscoelastic rod modeled according to the generalized Kelvin-Voigt behavior was studied in [229]. The choice of $\phi_{M}=\delta \left(\gamma \right)+a\delta \left(\gamma -\alpha \right)$ and $\phi_{\kappa }=EI\left(\delta \left(\gamma \right)+b\delta \left(\gamma -\alpha \right)+c\delta \left(\gamma -\beta \right)\right)$, which is a generalization of the standard Zener model, was proposed in [235] and used in [245] to study the lateral vibrations of viscoelastic rod. DO models were also used to analyze the influence of viscoelastic foundations on the dynamic stability of local and nonlocal rods [246]. Similarly, Varghaei et al. [247] investigated the nonlinear vibration of viscoelastic beams using a generalized Kelvin–Voigt model implemented via DO derivatives. Finally, Duan and Chen [248] investigated oscillatory shear flow between two parallel plates using DO form of the constitutive law for for viscoelastic fluids. Different effects of viscoelasticity over the structural response can be realized thanks to the generality of the DO models of viscoelasticity by employing specified choices for constitutive parameters. For instance, different viscoelastic constitutive models were employed in a study over the damping influence on the propagation of an initial Dirac delta disturbance through an infinite media. This provides the necessary foundation for designing an optimized damper as in [239].

## 5. Applications to Transport Processes

**Figure 5:**
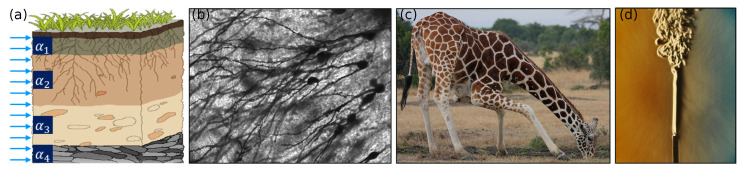
(**a**) Underground aquifers contain heterogenous layers of soils where each layer is characterized by a different level of porosity. The diffusion of groundwater through this multifractal media can be better described by DO operators, by replicating (mathematically) the parallel action of the different porous media in the order-distribution (see Section 5.3). Additional examples of multifractal systems where transport processes are better described via DO operators: (**b**) the diffusion of ions in neuronal dendrites [211], (**c**) the diffusion of pigments to form patterns in animals (see Section 5.2), and (**d**) turbulent flows. The subfigures (**a**–**d**) are taken from Wikipedia.

Several experimental investigations have shown that transport processes in many classes of materials are often characterized by anomalous mechanisms exhibiting either memory effects over various temporal scales or nonlocal effects over several spatial scales [249,250,251]. A direct consequence of this, as an instance, is a loss of the scaling invariance (CO or VO) noted in classical transport processes. Consequently, such processes cannot be modeled by using CO (integer or fractional) or even VO differential equations, as CO and VO diffusion equations lead to self-similar probability densities with a characteristic displacement exhibiting spatio-temporal scaling. The loss of the spatio-temporal scaling is a direct result of the presence of a spectrum of temporal or spatial scales in the transport process. The presence of several temporal scales, as an example, can be the result of the presence of a mixture of delay sources of variable strength [252] while the presence of distributed spatial scales can occur in transport through multifractal materials [211,215,253] (see Figure 5). Real-world examples of such complex transport processes include applications in geophysical and atmospheric phenomena [254,255,256,257], financial markets [258], turbulence [259], and even biology and medicine [211]. As discussed in Section 3, DODEs are very well suited to model such non-scaling anomalous transport processes exhibiting effects over multiple temporal and/or spatial scales.

From a thorough review of the literature it appears that anomalous diffusion, among other types of anomalous transport processes, has seen the maximum applications of DOFC. Therefore, we start by reviewing the application of DO models to complex diffusive transport processes, and then move on to other processes including reaction–diffusion, advection–diffusion, and hybrid propagation. In an effort to keep this review contained and focused on the main applications of DOFC to physical modeling, we present the key aspects and mathematical characteristics of the use of DODE in the modeling of transport processes. The interested reader can find extensive mathematical details on the implementation of DO transport models in [54].

### 5.1. Anomalous Diffusion Processes

As highlighted previously, diffusion processes in several classes of media exhibit strong anomalies wherein the mean square displacement (MSD) is not characterized by a definite (or unique) scaling exponent, [260,261,262,263]. As an example, the MSD in several systems grows as a power of the logarithm of time (*strong anomaly*) and shares the interesting property that the probability distribution of the particle’s position at long times is a double-sided exponential [261,262,263,264]. More specifically, the MSD varies as

(16) $$\langle x^{2}\left(t\right)\rangle \propto \log^{\nu }t$$

where ν is a positive constant. These diffusion processes are indicated as ultraslow diffusion (or, sometimes, superslow diffusion) processes and they do not conform to self-affine random processes. The most commonly referred example of such a strong anomalous diffusion process is the Sinai diffusion (ν=4) in which the particle moves in a quenched random force field [265]. Additional examples of such ultraslow diffusion behavior include polymer physics [266], numerical experiments on an area-preserving parabolic map on a cylinder [267], motion in aperiodic environments [268], and in a family of iterated maps [269]. We highlight that, apart from ultraslow diffusion, there exist other strong anomalies including retarding subdiffusion and accelerating subdiffusion, as well as retarding superdiffusion and accelerating superdiffusion. The specific form of the DO governing equation suitable to model either phenomena depends entirely on two factors: (1) the use of time and/or space-fractional DO derivatives, and (2) support of the strength function corresponding to the time- and/or space-fractional DO derivative. In the following, we will review the different modeling possibilities arising from combinations of the aforementioned factors.

In a series of seminal studies, Chechkin et al. [261,270,271] developed a DO framework for strongly anomalous diffusion mechanisms. They considered the time-fractional DO diffusion equation:

(17) $$\int_{0}^{1}\tau^{\beta -1}\phi \left(\beta \right)D_{t}^{\beta }c\left(t,x\right)\;d\beta =\bar{D}D_{x}^{2}c\left(t,x\right)$$

where c(t,x) denotes the particle concentration, and $\bar{D}$ denotes the diffusion coefficient. τ is a positive constant representing a characteristic time of the problem, and the strength function was chosen as $\phi \left(\beta \right)=\nu \beta^{\nu -1}$. The normalization condition for ϕ(β) on [0,1], i.e., $\int_{0}^{1}\phi \left(\beta \right)d\beta =1$ assumes v>0. As established in [261], this choice of ϕ(β) leads to ultraslow kinetics. More specifically, for the above mathematical setup, the MSD is obtained as

(18) $$\langle x^{2}\left(t\right)\rangle \propto \left\{ \begin{array}{cc} \frac{2\bar{D}}{\nu }t\log\left(\tau /t\right) & t/\tau \ll 1\\ \frac{2\bar{D}}{\Gamma (1+\nu )}\tau \log^{\nu }\left(t/\tau \right) & t/\tau \gg 1 \end{array}\right.$$

As evident, strong diffusion anomalies are described within the above DO diffusion formalism. In fact, it appears that the DODE in Equation (17) describes a subdiffusion random process which is subordinate to the Wiener process with a diffusion exponent decreasing in time (*retarding subdiffusion*). The same behavior was further highlighted by demonstrating that the modes of the solution, obtained via separation of variables, show an ultraslow, logarithmic, decay pattern. The waiting times (ψ(t)) of the diffusing particles corresponding to this setup are [271]

(19) $$\psi \left(t\right)\propto \frac{1}{t{\left[\log\left(t/\tau \right)\right]}^{1+\nu }}$$

and they do not have finite moments. Clearly, the DO diffusion equation can be interpreted as a limit of the continuous time random walk (CTRW) model with an extremely broad waiting-time probability density function (PDF), so that there are no finite moments [271].

We highlight that several authors have also analyzed the diffusion characteristics obtained via discrete order distributions [272,273,274] as well as a uniform strength distribution [261,272,273,274]. For the discrete time-fractional DO with $\phi \left(\beta \right)=\phi_{1}\delta \left(\beta -\beta_{1}\right)+\phi_{2}\delta \left(\beta -\beta_{2}\right)$ ($0<\beta_{1}<\beta_{2}\leq 1$, $\phi_{1}>0,\phi_{2}>0$, and $\phi_{1}+\phi_{2}=1)$, the characteristic displacement grows initially as $t^{\beta_{2}}$, whereas at large times it grows as $t^{\beta_{1}}$ indicating slow yet power-law growing diffusion. For the uniform strength function, that is ϕ(β)=1, the MSD is given as

(20) $$\langle x^{2}\left(t\right)\rangle \propto \left\{ \begin{array}{cc} 2\bar{D}t\log\left(\tau /t\right) & t/\tau <<1\\ 2\bar{D}\tau \log\left(t/\tau \right) & t/\tau >>1 \end{array}\right.$$

It appears that the DODE with the uniform strength function leads to slightly anomalous superdiffusion at small times, and to ultraslow diffusion at large times.

Another example of strongly anomalous diffusion processes corresponds to accelerating superdiffusion wherein the MSD, similar to ultraslow diffusion, does not exhibit a unique spatio-temporal scaling. In this class of diffusion processes, the diffusion exponent increases with time. Such processes are characterized using the following space-fractional diffusion equation [261],

(21) $$D_{t}^{1}c\left(x,t\right)=\int_{0^{+}}^{2}l^{\alpha -2}\bar{D}\;\Phi \left(\alpha \right)D_{x}^{\alpha }c\left(x,t\right)\;d\alpha$$

where *l* is dimensional positive constant. In [261], the authors obtained the MSD behavior by considering a two-term space-fractional diffusion equation, that is by choosing the strength function to be $\Phi \left(\alpha \right)=\Phi_{1}\delta \left(\alpha -\alpha_{1}\right)+\Phi_{2}\delta \left(\alpha -\alpha_{2}\right)$ with $0<\alpha_{1}<\alpha_{2}\leq 2$. For this DO diffusion equation, it was shown that at small times the characteristic displacement grows as $t^{1/\alpha_{2}}$, whereas at large times it grows as $t^{1/\alpha_{1}}$; clearly exhibiting superdiffusion with acceleration. The fundamental solutions for this discrete order distribution can be found in [275]. Exact solutions for a triple-order discrete distribution can be found in [276]. Random walk models corresponding to the space-fractional DO diffusion equation are presented in [275,277].

Notably, independently of the specific nature of the DODE (space-fractional or time-fractional) as well as of the strength function, the DO diffusion model no longer exhibits self-similarity or scale invariance. This is a direct result of the fact that the DO derivative modifies the constant- or even variable-order formulation, by integrating all possible orders over a certain range. The resulting solutions exhibit memory and/or nonlocal effects over several temporal and/or spatial scales leading to strong anomalities.

Building upon the time- and space-fractional DO diffusion models presented in Equations (17) and (21), several authors [278,279,280] developed DO diffusion models that lead to accelerating subdiffusion and retarding superdiffusion contrary to retarding subdiffusion and accelerating superdiffusion obtained via Equations (17) and (21), respectively. These DO diffusion models are given as [278,279,280]

(22a) $$D_{t}^{1}c\left(x,t\right)=\int_{0}^{1}\phi \left(\beta \right)\bar{D}D_{t}^{1-\beta }\left[D_{x}^{2}c\left(x,t\right)\right]d\beta$$

(22b) $$\int_{0}^{2}\phi \left(\alpha \right)l^{2-\alpha }D_{\left|x\right|}^{2-\alpha }\left[D_{t}^{1}c\left(x,t\right)\right]d\alpha =\bar{D}D_{x}^{2}c\left(x,t\right)$$

A direct comparison of the above equations with Equations (17) and (21) indicates an exchange in the presence of the time- and space-fractional DO derivatives, resulting in a class of mixed spatio-temporal DO derivatives. The detailed expressions of the MSD of the particles described via the above equations can be found in [278,279,280]. The MSD obtained via these formulations indicates that the anomalous diffusion phenomena described via Equation (22a) and Equation (22b) exhibit accelerating subdiffusion and retarding superdiffusion, respectively; that is, they become less anomalous in the course of time. Additional details on these anomalous behaviors are provided in the following. The DO time-fractional diffusion equation (Equation (22a)) describes a subdiffusion process which becomes less subdiffusive or, in other words, more classical in the course of time. The MSD demonstrates the occurrence of a transition from a growth characterized by a smaller exponent to a growth with a larger exponent. Equivalently, the probability density for a particle to remain around the origin exhibits a transition from slow to a faster decay. We highlight here that the fundamental solution for a discrete form of the Equation (22a), considering an infinite domain, can be found in [281]. The DO space fractional diffusion equation (Equation (22b)) describes power-law truncated Lévy flights, that is, a random process showing a slow convergence to a Gaussian, but exhibiting Lévy-like behavior at short times. This behavior manifests itself in the non-Gaussian Lévy scaling of the probability density to stay at the origin and in superdiffusive behavior. At short times, the central part of the PDF has a Lévy-stable shape, whereas the asymptotics decay with the power-law, faster than the decay of the Lévy-stable law. At long times, the central part of the PDF approaches the classical Gaussian shape, however, the asymptotics decay with the same power-law.

In addition to the above studies, several researchers have demonstrated the suitability of DOFC for modeling strongly anomalous diffusion behavior, particularly ultraslow diffusion, via stochastic descriptions [215,282,283,284,285,286,287]. Meerschaert et al. [282,288] developed a stochastic model based on random walks with a random waiting time between jumps. Scaling limits of these random walks are subordinated random processes whose density functions solve the DO ultraslow diffusion equation. Ultraslow diffusion has also been modeled using Langevin stochastic representations in [217,253,284,289]. As shown in [284], the solutions of DO Langevin equations have MSDs which describe retarding subdiffusion and ultraslow diffusion with logarithmic growth. Ultraslow diffusion is also obtained via the wait-first and jump-first Lévy walk models, which underlie the fractional dynamics involving DO material derivatives [289]. The approach in [289] is based on a strongly coupled CTRW, with the distribution of waiting times displaying ultraslow (logarithmic) decay of the tails. Similarly, the authors of [283,285] obtained the space-fractional DO diffusion formulation as the continuum limit of a random process which is characterized by the presence of a distribution of spatially-dependent jumping rate and the Lévy distributed jumping size. As described in [283,285], such a system is well suited to describe diffusion in multifractal systems which do not possess a unique Hurst exponent and, consequently, exhibit a lack of scaling. The lack of scaling in multifractals requires a generalization of stochastic Lévy equation by admitting a spectrum of the Lévy index. The continuum limit of this stochastic equation is the DO diffusion equation. A detailed mathematical analysis of the Lévy models is presented in [286] and a Lévy mixing based probabilistic interpretation of the DO diffusion model is presented. The characteristics of the model are exemplified by a direct application to slow diffusion, particularly the delayed Brownian motion. A similar stochastic representation, given in the form of the Brownian motion subordinated by a Lévy process was to model accelerating subdiffusion in [290]. Additionally, the authors of [290] also constructed an algorithm for computer simulations of accelerating subdiffusion paths via Monte Carlo methods.

Before proceeding further, we briefly review the contributions that several researchers made to the different mathematical aspects of the DO diffusion equations. Exact solutions corresponding to Dirichlet, Neumann, and Cauchy boundary conditions for the time-fractional DO diffusion Equation (17) can be found in [291]. The fundamental solution of the DODE corresponding to a uniform strength distribution can be found in [272,273,274]. Mainardi et al. [292] obtained the fundamental solution of the time-fractional DO diffusion equation based on its Mellin–Barnes integral representation. They also presented a series expansion of the fundamental solution that clearly highlights, within the solution, the presence of several time-scales related to the distribution of the fractional-orders in the DO diffusion equation. Asymptotic solutions to initial and boundary value problems based on the DO time-fractional diffusion equations can be found in [293,294]. Some additional and important mathematical aspects, such as the existence of the solution to different types of DO diffusion equations, the solvability of DO diffusion equations, subordination properties, and positivity of the solution were addressed in [59,63,263,287,295,296,297,298,299,300]. In a series of papers [71,72,301], Luchko analyzed the well-posedness of the DO formulation via maximal principles, and obtained *a priori* norm estimates for solutions to both linear and nonlinear DO diffusion equations. Luchko has also provided a survey of these maximal principles in [302]. Further, the well-posedness of the inverse problem, that is the determination of the strength distribution of the DO and its support, has been analyzed in detail in [303,304,305,306,307]. The analysis of the well-posedness of the inverse problem is highly essential to promote applications of DOFC since it determines whether the DO framework is suited to model a given real-world application. In other terms, given a set of experimental or real-world data, the analysis of the inverse problem determines whether DOFC is well suited to model the dataset and hence, it also indicates if the corresponding system exhibits multiscale (temporal and/or spatial) characteristics.

The remarkable properties of the DO diffusion formalism provided a strong foundation for the development of other DO transport formulations: DO reaction–diffusion, DO advection–diffusion, and DO wave propagation. Before reviewing these other applications, we briefly overview some recent, yet remarkable, real-world applications of the DO diffusion formulation (see Figure 5). Grain boundary diffusion in engineering materials at elevated temperatures, that often determines the evolution of microstructure, phase transformations, and certain regimes of plastic deformation and fracture, was modeled via a DO diffusion framework in [308]. DO diffusion equations have also been used to model the diffusion of mobile ions in different electrolytic cells [309,310,311]. The predictions of the DO model closely matched experimental data which indicated the presence of different diffusive regimes. A similar application was presented in [312], where DO operators were introduced into the Letokhov model of photon diffusion to model non-resonant random lasers. Very recently, the effect of disordering of nanotubes within an electrode, on the impedance of a supercapacitor, was modeled using the DO subdiffusion model in [313]. All these applications highlighted the ability of the DO diffusion formulation to accurately capture highly anomalous diffusion behavior arising out of the presence of multiple temporal and/or spatial scales.

### 5.2. Reaction–Diffusion Processes

An interesting application of DOFC involves the modeling of reaction–diffusion systems. Reaction–diffusion processes describe changes in the concentration of interacting chemical substances both in space and time. Reaction–diffusion processes have been linked to the formation of spots and patterns in different animals and birds [314,315], among many other real-world applications [125,316] (see Figure 5c). Distributed-order derivatives help to account for the heterogeneity and multifractal nature of the diffusing medium, typical of these applications. More importantly, the DO derivatives also account for the multiple sources of the reacting chemicals within the heterogeneous system. This allows for compact yet more comprehensive theoretical formulations of the reaction–diffusion mechanisms when compared to classical integer-order based approaches. Several authors have analyzed complex reaction–diffusion systems using DO derivatives [102,129,149,316,317]. Detailed mathematical formulations along with closed form solutions for DO reaction–diffusion equations can be found in [316,318]. The effect of different strength functions as well as the specific nature of the DO reaction–diffusion equation was analyzed numerically in [102,129,149]. Very recently, Guo et al. [148] analyzed a 3D Gordon-type reaction–diffusion model of colliding and diffusing Gordon-type solitons. The numerical results provided a deeper understanding of the complicated nonlinear behavior of the 3D Gordon-type solitons system while highlighting the remarkable capabilities of the DO derivatives in capturing the collision and diffusion of the solitons.

### 5.3. Advection-Diffusion Processes

The VO diffusion equation formed the basis of several interesting investigations involving strongly anomalous advection-diffusion processes in complex systems, particularly those related to hydrology such as, for example, geomigration [319], transport of solutes in heterogeneous media [257,320], the spread of contaminants in groundwater [321], as well as groundwater flow [322]. Indeed, several theoretical and experimental studies have shown that the transport of fluids and pollutants through geological aquifers exhibits the presence of multiple spatio-temporal scales arising from the multifractal nature of the aquifers. The multifractality is a direct consequence of the porous, fractured, layered, and heterogeneous nature of the aquifers (see Figure 5a). The underlying distinctive characteristics of DOFC make it a very well suited modeling approach for the aforementioned anomalous transport phenomena experienced in hydrology.

The detailed mathematical analysis of a DO advection-diffusion equation with a discrete distribution of orders was presented in [77]. Analytical solutions were obtained in [77] for a time- and space-fractional formulation and some interesting derivations including the spectral representation of the fractional Laplacian operator were presented. Later, several researchers used DOFC to model advection–diffusion in complex problems, particularly those related to hydrology. A DO advection–diffusion model was proposed in [256] to model infiltration, absorption, and water exchange in mobile and immobile zones of swelling soils. A similar formulation was adopted in [319] to model a geomigration process in a geoporous medium saturated with a salt solution that exhibits subdiffusive characteristics. Several researchers also used DOFC to model subdiffusive characteristics observed in the transportation of solutes in heterogeneous porous media [257,320,323]. Very recently, an interesting application of DOFC was proposed to simulate superdiffusion of dissolved phase contaminants in groundwater [321]. In this study, several insights including the specific impact of different geometric properties of the contaminants on their spatial distribution pattern, were derived using the DO advection-diffusion model.

### 5.4. Wave Propagation

Several authors investigated DO models for wave propagation by directly extending the DO diffusion approaches reviewed in Section 5.1. More specifically, this process involved altering the support of the strength function corresponding to the DO time-fractional derivative from [0,1] to an interval within [1,2]. The most generalized versions of the one-dimensional DO wave equation can be obtained by modifying Equations (17) and (21) as

(23a) $$\int_{1}^{2}\tau^{\beta -1}\phi \left(\beta \right)D_{t}^{\beta }u\left(t,x\right)\;d\beta =E_{0}D_{x}^{2}u\left(t,x\right)$$

(23b) $$D_{t}^{2}u\left(x,t\right)=\int_{0^{+}}^{2}l^{\alpha -2}E_{0}\Phi \left(\alpha \right)D_{x}^{\alpha }u\left(x,t\right)\;d\alpha$$

where u(x,t) denotes the particle displacement and $E_{0}$ denotes a material constant. A different set of DO wave equations can be obtained by modifying the support of the strength function and using mixed spatio-temporal DO derivatives, similar to Equations (22a) and (22b). The qualitative discussions on the application of DO models for multifractal systems, presented for other types of transport processes reviewed in this Section 5, also holds for DO wave propagation. As an example, the propagation of elastic waves through dissipative media exhibiting multifractal viscoelastic behavior (see Section 4) is described via time-fractional DO models [221,324]. Similarly, elastic wave propagation via attenuating media characterized by simultaneous microstructural and nonlocal (hence, multiscale) effects can be described via space-fractional DO models [213]. Important mathematical aspects such as the existence and uniqueness of the solution to the DO time-fractional wave equation have been outlined in detail in [63,325,326,327]. Additionally, the fundamental solutions of the DO wave equation have been derived in [298,325,327,328] using the technique of the Fourier and Laplace transforms. Numerical experiments highlighting the specific effects of the DO model parameters have been used to derive interesting insights into the DO wave equation in [298,325,328].

Another possible route to develop the DO wave propagation formulation consists in formulating DO stress–strain constitutive relations within the classical elastodynamic problem as proposed in [324,329]:

(24) $$\sigma =E_{0}\int_{0}^{1}\phi \left(\beta \right)D_{t}^{\beta }\varepsilon d\beta$$

This approach resembles the formulation of DO viscoelastic models (see Section 4) and indeed leads to a hybrid propagation model that also captures dissipation. The DO wave propagation model was then used to simulate the interaction of compressional waves with an interface separating two dissimilar media. Further, the impact of the support and definition of the strength function were analyzed on the wave scattering at the interface.

## 6. Applications to Control Theory

In this section, we analyze the applications of DOFC to control theory. The foundation as well as motivation for the application of DOFC to control theory follows from a successful application of COFC to model complex control phenomena. The use of CO fractional controllers has enabled robust control and helped achieving highly desirable dynamic control characteristics. A detailed review of theory and applications of COFC in control theory can be found in [36]. In this regard, recall that a fractional derivative implicitly embeds within itself time-delays, or in other terms, it accounts for the memory of past events. Consequently, the presence of a distribution of fractional-order derivatives translates, physically, to the presence of a mixture of delay sources (similar to what is discussed in Section 5). These DO characteristics have helped achieve high performance controllers with several applications ranging from secure messaging [330], to control of motors [331,332] as well accurate frameworks to model robust stability of gene regulatory networks [332]. Broadly speaking, the applications of DOFC to control theory can be divided into two categories: (1) the development of DO controllers and (2) study of the stability and control of DO systems; the majority of the studies being focused on the latter category. In the following, we first review the DO controllers and their applications, before considering their stability. A few other studies have numerically analyzed various DO system identification techniques [220,333] and DO optimal control problems [100,334]. However, the basic DO control theory employed in the latter studies are derived from the two broad categories mentioned above.

### 6.1. DO Controllers and Filters

Several theoretical and experimental studies have shown that fractional-order designs can enhance both the flexibility and robustness of the controllers as a result of the additional parameters represented by the fractional-orders themselves. Tuning of the fractional-orders allows for superior control characteristics. As an example, consider the CO PID controller PI${}^{\lambda }$D${}^{\mu }$. The value of the order λ in PI${}^{\lambda }$D${}^{\mu }$ control affects the slope of the low frequency range of the system as well as the peak value of the system. On the other hand, the value of the order μ affects the accuracy of the dynamic closed-loop response, the system overshoot, and the stability. For a more detailed discussion of the roles of λ and μ, the interested reader is referred to the work in [36]. It is immediate that a distribution of several CO controllers can lead to highly accurate and robust control. In fact, DOFC allows the development of a highly generalized controller from which all other types of controllers (such as, for example, the classical integrator and differentiator, the classical PID, and the fractional PI${}^{\lambda }$D${}^{\mu }$) can be recovered.

**Figure 6:**
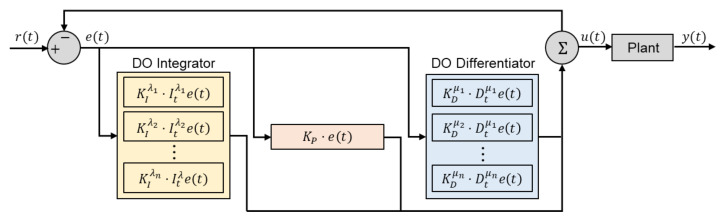
Block diagram illustrating the feedback DO controller based on Equation (25). The fractional-orders $\mu_{k},\lambda_{k}\in \left(0,1\right]$. This is a highly general controller from which all classical, CO, and DO controllers, as well as the DO PID controller can be recovered by an appropriate choice of the controller constants. As an example, the DO differentiator can be obtained by setting $K_{I}^{\lambda_{k}}=0$, $K_{P}=0$, and $K_{D}^{\mu_{k}}\neq 0$. As evident, the DO differentiator consists of a network of CO differentiators. Similarly, the DO PID controller would require that $K_{P}\neq 0$, $K_{D}^{\mu_{k}}\neq 0$ and $K_{I}^{\lambda_{k}}\neq 0$.

In the most general form, the transfer function of a DO controller can be expressed as [36]

(25) $$G\left(s\right)=\int_{\beta_{1}}^{\beta_{2}}\phi \left(\beta \right)\frac{1}{s^{\beta }}d\beta$$

where *s* is a complex variable. The interval $\left[\beta_{1},\beta_{2}\right]$ dictates the specific nature of the controller. Note that a DO low-pass filter can be obtained from the DO controller via the transformation s→T(β)s+1 [335]. The above formulation is highly general in the sense that all the classical, CO, and DO controllers can be recovered from the same by an appropriate choice of the strength function. As an example, the classical integrator can be obtained by choosing ϕ(β)=δ(β−1), the classical differentiator can be obtained from ϕ(β)=δ(β+1), the classical PID from $\phi \left(\beta \right)=k_{P}\delta \left(\beta \right)+k_{I}\delta \left(\beta -1\right)+k_{D}\delta \left(\beta +1\right)$ ($k_{P},\;k_{I}\;\mathrm{and}\;k_{D}$ are constants to be tuned), the fractional PID from $\phi \left(\beta \right)=k_{P}\delta \left(\beta \right)+k_{I}\delta \left(\beta -\lambda \right)+k_{D}\delta \left(\beta +\mu \right)$, and so on. It is immediate to see that a DO PID controller can be also obtained directly from the controller in Equation (25), by insisting that the support of the weight function lies within the interval [−1,1]. DO PID controllers have been studied in detail in a series of papers by Jakovljević et al. [336,337,338]. Note that in the case of a DO controller, the strength function in Equation (25) can have infinite support. In fact, as established in [339], any DO controller can be developed by appropriate composition of the DO integrator ($0\leq \beta_{1}<\beta_{2}\leq 1$), the classical integrator (1/s) and the classical differentiator (s). The different DO controllers have been schematically illustrated in Figure 6.

The impulse response and asymptotic behavior of the DO controllers have been derived in [335,340]. Additionally, a physical realization of the DO integrator using a series of capacitors has been developed in [210,340]. The DO controllers have been applied to control motors [338] and robots [331] among many other applications [36]. As observed in these studies, the DO controllers reduce the maximum overshoot while guaranteeing a fast dynamic response and a zero steady-state error [36,336,337,338]. Furthermore, the phase curves of DO PID controllers are non-constant and much wider than the corresponding CO controllers making them more robust to system uncertainties [331]. Therefore, the DO controllers exhibit unique frequency response characteristics, and provide highly robust and accurate control.

### 6.2. Stability and Control of DO Systems

The development of robust and accurate DO controllers prompted several researchers to analyze the stability of both linear and nonlinear DO dynamical systems. Most of the studies conducted on linear systems correspond to the bounded-input bounded-output (BIBO) stability analysis of DO linear time-invariant (LTI) systems. On the other hand, the nonlinear studies have focused primarily on the Lyapunov stability of the equilibrium points of the DO system. First, we briefly review the key highlights of the DO LTI systems and their applications. Consider a DO system described via the following LTI DODE and algebraic output equation,

(26) $$\begin{array}{c} \int_{0}^{1}\phi \left(\beta \right)D_{t}^{\beta }x\left(t\right)d\beta =Ax\left(t\right)+Bu\left(t\right)\\ y(t)=Cx(t)+Du(t) \end{array}$$

where x(t) is the state vector, u(t) indicates the input, and y(t) indicates the output of the system. *A*, *B*, *C*, and *D* are matrices of appropriate dimensions. Note that the interval of the DO derivative in the above single-input single-output (SISO) system can be converted to a more general interval $\left[\beta_{1},\beta_{2}\right]\in \left[0,1\right]$. Applying a set of Laplace and inverse Laplace transform to the above DODE with the assumption that x(0)=0 and u(t)=δ(t), the following expression can be obtained,

(27) $$x\left(t\right)=L^{-1}\left[\underset{G(s)}{\underset{︸}{{\left[\left(\int_{0}^{1}\phi \left(\beta \right)s^{\beta }d\beta \right)I-A\right]}^{-1}}}B\right]\left(t\right)$$

where *I* denotes the identity matrix. As established in [341,342,343], the DO LTI system in Equation (26) with the transfer function H(s)=CG(s)B+D is BIBO stable *iff* all the roots of the secular equation corresponding to |G(s)I−A|=0 have negative real parts. The contours of this stability region have been derived based on the latter principle for different definitions of the strength function in [342,344]. The stability contours are often impossible to express via elementary functions, which makes the stability tests of DO systems more complicated than their constant- and integer-order counterparts. In this regard, the Lagrange inversion theorem was utilized in [345] to obtain explicit expressions for the stability contours. Several interesting properties of these stability curves such as the slope of the tangent at very high and very low frequencies, convexity, inability to cut itself, location in the first and fourth quadrants, and shifting and enhancement of the area of the stability via multiplication of suitable functions to the strength distribution, have been presented in [346,347,348].The above mentioned properties of the stability boundaries were used in [347] to present a remarkable framework for the robust stability analysis of DO LTI systems with uncertain strength distributions and dynamic matrices. More specifically, these properties were used to show that the stability boundary of DO LTI systems can be accurately located in a certain region on the complex plane defined by the upper and lower bounds of the strength distribution. These results are sufficient to ensure robust stability in DO LTI systems with uncertain strength functions and uncertain dynamic matrices. The above framework presented in [347] is highly relevant for real-world applications that are commonly accompanied by uncertainties. Additional discussions on the stabilization, controllability, and passification of DO LTI systems can be found in [349,350,351,352].

The DO LTI framework discussed above has been used to analyze different systems: the solar wind-driven magnetosphere ionosphere system (a complex driven-damped dynamical system which exhibits a variety of dynamical states) [341,348], a DO Lotka–Volterra predator–prey system (a system with multiple time-delays) [353], the DO Chen system [354], and gene regulatory systems [332]. All the aforementioned applications differ primarily in the choice of the strength function which directly affects the stability and control of the system.

In nonlinear systems, researchers have focused mainly on analyzing the Lyapunov stability of systems, as also mentioned previously. The Lyapunov direct method, used for analysis of stability, was first generalized for nonlinear time-varying DO systems in [355,356,357] and was used to determine the stability or asymptotic stability of certain nonlinear systems including a DO analog of the Lorenz system. The theoretical framework proposed in the studies [355,356] was then used to analyze different nonlinear time-varying DO systems including a DO consensus model [358], the DO Lorenz system [359], and the DO Van der Pol oscillator [330,360]. The consensus of multi-agent systems with fixed directed graphs and described by DODE, was analyzed in [358] and sufficient conditions were obtained for robust consensus in the presence and absence of external disturbances. Recently, the stability and control of a DO Van der Pol were analyzed in [330], wherein the intervals of the different model parameters at which this oscillator exhibits periodic, chaotic, and hyperchaotic behaviors, were calculated using Lyapunov exponents. Further, a robust scheme was presented in [330] to achieve complete synchronization between two DO hyperchaotic unforced Van der Pol oscillators. This synchronization allowed the development of a secure messaging system for a text which contains alphabets, numbers, and symbols.

## 7. Conclusions

This paper presented an overview of the general area of Distributed-Order Fractional Calculus (DOFC) with particular focus on its applications to scientific modeling of complex systems. A branch of the broader field of fractional calculus, DOFC has rapidly emerged and captured the attention of many researchers in science and engineering. This rapid growth was mostly due to its remarkable ability to capture complex multiscale processes. Phenomena like multiple relaxation times in viscoelasticity, multiple temporal and spatial scale effects in transport processes, and mixture of time delays in control theory, just to name a few, have all illustrated the significant performance of DOFC over more traditional integer-order techniques. The main goal of this review was to provide a snapshot in time of the field of DOFC and to guide the interested reader into an introductory journey through this fascinating topic. In this regard, we highlight that the content of technical papers was only briefly addressed in order to favor a more general discussion of the evolution of the field in its different areas of application.

Despite the recent substantial growth in DOFC research, there are still many areas holding significant opportunities for further development. While some preliminary work is available on distributed-variable models, a comprehensive framework for distributed-variable-order fractional calculus (DVOFC) is still lacking. A key factor that adds to the complexity of formulating DVOFC is the existence of different definitions for VO operators that exhibit different memory characteristics. Thus, a unified definition of the different variable- and distributed-order operators and an analysis of their mathematical properties would certainly be beneficial. In these operators, the order-variation can be a function of different dependent or independent physical variables (such as, for example, temperature, space, time, and energy). The combination of the DO and VO formalisms should allow the simulation of highly complex physical systems which are both evolutionary (therefore, requiring VO operators) and multifractal (requiring DO operators) in nature. Another possible extension of currently available DO operators follows from the use of normalized self-similar strength functions within the definition of DO operators, which can be considered analogous to random-order operators. Particularly lacking is a rigorous mathematical analysis of the properties of such operators. Despite the above challenges, the extension of DOFC to these areas can have important applications in modeling random and chaotic dynamics observed, as an example, in turbulent dynamics, noise and vibration control, or even in financial systems. These models could even form the basis for the development of highly accurate risk analysis and control models.

It should be pointed out that, despite the rapidly growing number of related studies, there are still several open questions that need to be addressed before DOFC could become a mainstream modeling approach for common real-world applications. A critical step to promote the broader use of DOFC models is to establish the connection between the mathematical properties of DO operators (i.e., the strength function and its support) and the physical properties and parameters of the system to be modeled. In other terms, the identification of closed form relations linking the mathematical parameters of the DO operators to the physical parameters of the system at hand are of paramount importance to foster the use of DOFC tools in scientific modeling.

## Data Availability

This article has no additional data.
